# Examining and addressing evidence-practice gaps in cancer care: a systematic review

**DOI:** 10.1186/1748-5908-9-37

**Published:** 2014-03-25

**Authors:** Jamie Bryant, Allison Boyes, Kimberley Jones, Rob Sanson-Fisher, Mariko Carey, Rae Fry

**Affiliations:** 1 Priority Research Center for Health Behavior, Health Behavior Research Group, University of Newcastle and Hunter Medical Research Institute. HMRI Building, University of Newcastle, Callaghan NSW, Australia; 2 Cancer Council New South Wales, Woolloomooloo NSW, Australia

**Keywords:** Evidence-based practice, Guideline adherence, Translational medical research, Medical oncology, Neoplasms

## Abstract

**Background:**

There is increasing recognition of gaps between best scientific evidence and clinical practice. This systematic review aimed to assess the volume and scope of peer-reviewed cancer research output in the years 2000, 2005, and 2010.

**Methods:**

Eligible papers were published in English and reported on evidence-practice gaps in cancer care. The electronic database Medline was searched for three time periods using MeSH headings and keywords. Abstracts were assessed against eligibility criteria by one reviewer and checked by a second. Papers meeting eligibility criteria were coded as data-based or non-data-based, and by cancer type of focus. All data-based papers were then further classified as descriptive studies documenting the extent of, or barriers to addressing, the evidence-practice gap; or intervention studies examining the effectiveness of strategies to reduce the evidence-practice gap.

**Results:**

A total of 176 eligible papers were identified. The number of publications significantly increased over time, from 25 in 2000 to 100 in 2010 (p < 0.001). Of the 176 identified papers, 160 were data-based. The majority of these (n = 150) reported descriptive studies. Only 10 studies examined the effectiveness of interventions designed to reduce discrepancies between evidence and clinical practice. Of these, only one was a randomized controlled trial. Of all data-based studies, almost one-third (n = 48) examined breast cancer care.

**Conclusions:**

While the number of publications investigating evidence-practice gaps in cancer care increased over a ten-year period, most studies continued to describe gaps between best evidence and clinical practice, rather than rigorously testing interventions to reduce the gap.

## Background

### The importance of reducing evidence-practice gaps in healthcare

Evidence-based practice is the ‘conscientious, explicit and judicious use of current best evidence in making decisions about the care of individual patients’ [1]. There is increasing recognition of gaps between best scientific evidence and clinical practice in many fields of healthcare [2-5]. It has been estimated that up to 40% of patients fail to receive treatments shown to be effective [4], while 20% to 25% receive treatments that are not needed, or potentially harmful [5]. Reducing the gap between best evidence and clinical practice is associated with reductions in patient morbidity and mortality [6-8], and reduced healthcare costs [9]. In the past ten years, increasing attention has been directed to addressing barriers to the translation of research into clinical practice to improve patient outcomes [10-12].

### Concern about evidence-based practice in cancer care

The past decade has been marked by increases in both cancer incidence and survival [13,14]. However, concern about disparities between best-evidence practice and cancer care has persisted for some time. In the 1999 report ‘Ensuring Quality Cancer Care’ [15], the US National Cancer Policy Board stated that ‘reasons for failure to deliver high-quality care have not been studied adequately’ (pg 4). The report made a number of recommendations, including that clinical practice guidelines be developed and implemented to ensure optimal care is provided to patients, and that the quality of care provided be measured and monitored [15]. Awareness of the need to address evidence-practice gaps in cancer care was further heightened by the landmark 2001 Institute of Medicine report ‘Crossing the Quality Chasm’, which identified that high rates of misuse, underuse, and overuse of health services have created a ‘chasm’ between best evidence and medical practice [16].

### Volume of research output as a measure of research effort

Given increased acknowledgement of the need to address evidence-practice gaps in cancer care, it might be expected that research efforts to ensure effective translation of knowledge into clinical practice would also have increased over this time period. Although not without limitations [17], examining the volume of peer-reviewed research output using bibliometric methods is a proxy indicator of scientific productivity [18,19]. Volume of research output provides information about the research capacity of a field, including the areas where research funding has been allocated, and where clinician and researcher effort have been directed.

### Research design as a measure of progression of research effort

While volume of research output provides an indication of the amount of work being conducted in a particular field, it fails to provide information about the type or quality of work. The effective translation of evidence into practice is complex and depends on a number of factors, one of which is the type of research evidence generated [20]. To advance evidence-based practice, a logical progression of research is required. First, there is need to examine whether there is a discrepancy between current practice and best-evidence practice, and if so, the magnitude of the discrepancy. If a gap is found that is likely to have clinically or economically important implications, research is then needed to develop evidence about how best to effectively reduce this gap. Methodologically rigorous intervention studies are critical to produce evidence about the most effective strategies for delivering best practice healthcare. Interventions might aim to educate clinicians [21], change the practice environment [22], or change contingencies [23].

In 2010, Evensen *et al*. [24] examined the number of published studies related to the evidence-practice gap across nine specific guidelines related to family medicine, three of which related to cancer. While a large number of studies were identified, only 15% of the studies were intervention studies and few met quality criteria for methodological rigor established by the Cochrane Effective Practice and Organization of Care (EPOC) Group [25]. Providing optimal care to the increasing population of cancer patients and survivors has been described as a public health challenge [26]. Translational step three (T3) of the National Institutes of Health Roadmap [27] argues that there is a need for research to examine translation of evidence generated into the clinical care provided to patients. However to date, there has been no examination of the research attention given to implementation of evidence into clinical care at the T3 level in the wider cancer literature.

### Aims

To examine in the cancer literature in the years 2000, 2005 and 2010:

1. The number of publications examining evidence-practice gaps;

2. The number of data-based versus non-data-based publications examining evidence-practice gaps, including the number describing evidence practice gaps compared to the number evaluating interventions to reduce evidence practice gaps;

3. The number of data-based publications examining evidence-practice gaps by cancer type and research design.

## Methods

### Inclusion and exclusion criteria

Eligible papers were those published in English in 2000, 2005, and 2010 that reported on evidence-practice gaps in cancer care. The year 2000 was selected as the starting point for this review given that the influential report by the National Cancer Board was published in 1999 [15]. Studies examining the effectiveness of treatments on cancer recurrence, disease-free survival, or overall survival were excluded, as were studies examining evidence-practice gaps for cancer screening, editorials, letters to the editor, dissertations, and protocol papers.

### Literature search

The electronic database Medline was searched using the OVID platform. The search strategy included three categories of search terms: guideline adherence/evidence based practice, cancer, and treatment types (full search strategy available in Additional file 1). Medline was selected as the database of choice given its focus on biomedicine and health publications in scholarly journals. Searches were restricted to English language publications and human studies. A Google Scholar search using combinations of the above keywords was also conducted to ensure relevant papers were not missed. A copy of the review protocol is available in Additional file 2.

### Paper coding

Retrieved abstracts were initially assessed against the eligibility criteria by one reviewer (JB) and rejected if the reviewer determined from the title and abstract that the study did not meet inclusion criteria. Full text copies of the remaining publications were retrieved and further assessed against eligibility criteria to confirm or refute inclusion. Papers meeting the eligibility criteria were then categorized as follows:

1. Data-based or non-data-based: Data-based publications were those reporting new data or new analysis of existing data. Non-data-based publications included review, commentary, discussion, or summary papers.

2. Cancer type: All data-based papers were classified according to the cancer type of the study sample according to the International Classification of Diseases for Oncology [28] and body system-specific cancer classification.

3. Research design: All data-based papers were further classified into one of the following categories: Descriptive studies using cross-sectional study designs to document or describe the evidence-practice gap or barriers to addressing the evidence-practice gap; Intervention studies using experimental designs to test strategies to reduce the evidence practice gap. Studies using an experimental design were also assessed as to whether the design was one of the four types allowed by the EPOC criteria- randomized controlled trials, clinical controlled trials, controlled before and after studies, or interrupted time series studies.

A random sample of 10% of papers identified as eligible was checked for relevance and double-coded by a second reviewer (MC).

### Analysis

Chi-Square tests for equal proportions were conducted to determine whether the total volume of research output changed over time, and whether the number of descriptive studies changed over time.

## Results

### Search results

**Figure 1:**
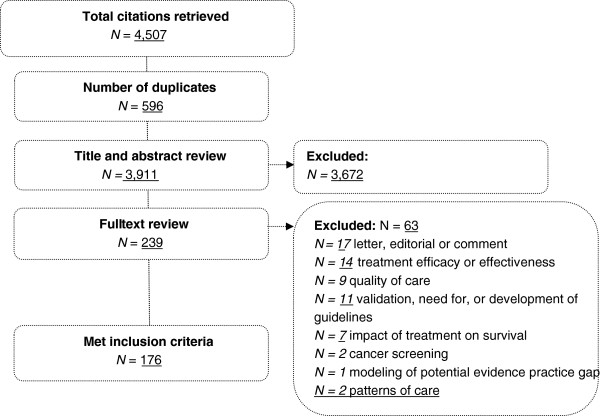
Flow chart of search strategy and study selection.

A total of 4,507 citations were retrieved using the key words. After assessment against the eligibility criteria and double coding by a second reviewer (with 100% agreement achieved), 176 relevant studies meeting the eligibility criteria were included in the review (Figure 1). A list of included citations is provided in Additional file 3.

### Volume of research output over time

**Figure 2:**
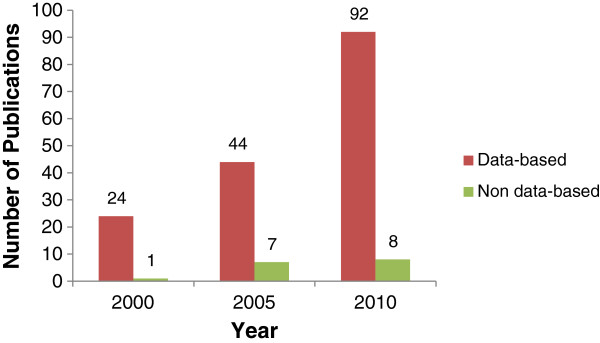
Number of publications addressing the evidence-practice gap in cancer care in 2000, 2005 and 2010.

Of the 176 relevant publications addressing evidence-practice gaps in cancer, 25 were published in 2000, 51 in 2005, and 100 in 2010 (see Figure 2). There was a significant increase in the total number of publications over time (p < 0.001).

### Number of data-based versus non-data-based publications

The number of data-based and non-data-based publications addressing evidence-practice gaps in cancer are reported in Figure 2. A total of 160 data-based and 16 non-data-based publications were identified.

### Number of data-based publications by cancer type

**Figure 3:**
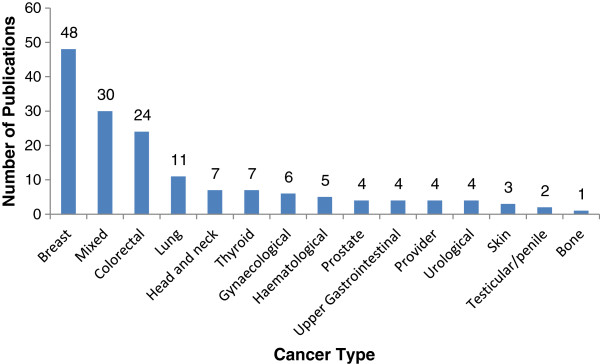
Distribution of data-based studies by cancer type (N = 160).

The distribution of data-based studies by cancer type is reported in Figure 3. The cancer type with the highest number of publications was breast cancer (48 publications), followed by studies with heterogeneous samples of cancer types (30 publications). Four papers examining provider behavior could not be coded by cancer type.

### Number of data-based publications by research design

**Figure 4:**
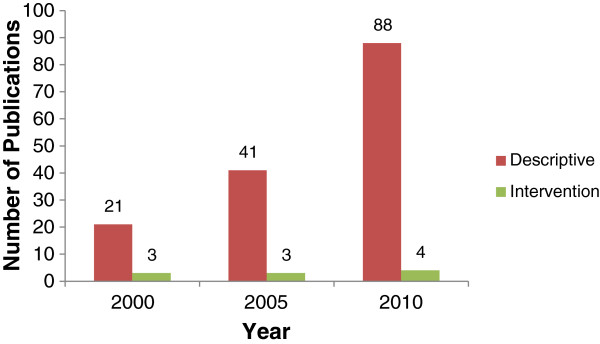
Number of descriptive and intervention studies addressing the evidence-practice gap in cancer care in 2000, 2005 and 2010.

Of the 160 data-based publications identified, the large majority (n = 150) used cross-sectional designs to describe evidence-practice gaps (Figure 4). Only ten studies reported on the effect of an intervention designed to reduce an evidence-practice gap. Of these, one used a randomized controlled design, one used a controlled before and after design, and the remainder used pre/post designs without a control condition. There was a significant increase in the number of descriptive studies over time (p < 0.001), however the number of intervention studies did not increase over time.

## Discussion

Reducing the gap between best evidence and clinical practice in cancer care will improve patient morbidity and mortality [6-8] and reduce healthcare costs [9]. This review identified a small volume of research in cancer care addressing this important issue. Of the 176 publications identified, the majority (91%) provided new data. Only 9% of publications were reviews, commentaries, or summaries of the existing evidence base. This is an encouraging finding and suggests that a substantial proportion of research effort is directed toward empirical work. However, further examination revealed that 94% of data-based publications were descriptive studies, with only ten intervention studies identified. The number of intervention studies did not increase over time. Studies most commonly focused on breast cancer care.

While descriptive research provides important information about current practice, it does not maximize research benefits by comparing the effectiveness of approaches to improve care. The small number of intervention studies, and in particular the small number that used rigorous evaluation designs, may be explained by the difficulty of carrying out well-controlled intervention trials in this field. Changes to clinical practice may require changes to processes of care, clinician knowledge, and organizational culture, so the unit of analysis for such studies is often the hospital, ward, or clinic. These types of system-focused interventions are often incompatible with randomized controlled designs where the unit of randomization is the individual [29]. An alternative to the traditional randomized controlled trial suitable for evaluating interventions involving organizational change is the cluster randomized controlled trial. However, this design poses complex logistical challenges, such as the need to obtain agreement for implementation from all clinicians within the cluster, difficulties obtaining a large enough sample of hospitals or clinics, and the substantial costs associated with evaluating the intervention across many sites [30]. Intervention studies also require multi-disciplinary collaboration and a specific repertoire of research skills, while descriptive research requires relatively less time and fewer resources. In a professional environment where both volume and impact of research output is valued, researchers may be more motivated to undertake descriptive work rather than complex and time intensive intervention studies, or focus their effort on randomized controlled trials of individual-level interventions. The design challenges may be overcome by the use of alternative research designs such as interrupted time series or multiple baseline designs, and by commitment from funding bodies to finance robust intervention trials. Targeted funding rounds that prioritize research on strategies to close the evidence-practice gap may also be important to increasing productivity in this area.

The small number of intervention trials may also be the result of a perception in clinical oncology that optimal cancer care is already being delivered to patients and, therefore, research to ensure translation of clinical research into practice is not needed. It is simplistic to presume, however, that new evidence is routinely integrated into clinical practice guidelines, policy, and then clinical care [31,32]. Changing established patterns of care is difficult, and often necessitates the involvement of cancer patients and their advocacy groups, individual practitioners, senior administrators of healthcare organizations, and policy makers [32]. Passive dissemination of evidence via clinical practice guidelines and publications produce only small changes in clinical practice, and there is insufficient evidence to show that such strategies have any effect on patient outcomes [33]. Further, uptake of new evidence is inconsistent even when active implementation strategies are undertaken [4,34]. For example, one descriptive study examined the impact of the American Society of Clinical Oncology (ASCO) guidelines regarding use of hematopoietic colony-stimulating factors (CSF) on cancer care. Six months after active dissemination and implementation of the guidelines, it found that only 61% of CSF prescriptions complied with ASCO guidelines [35]. In addition, the finding that 94% of data-driven papers identified in this review described evidence-practice gaps in cancer care highlights the importance of increasing implementation research effort in the field.

The finding that a large proportion of research effort has been directed toward breast cancer care is in accordance with previous findings that breast cancer research dominates the quality of life research field [36]. Prostate cancer is similar to breast cancer in terms of incidence [37], and other high-incidence cancers such as lung cancer and bowel cancer have higher mortality rates than breast cancer [37]. Therefore, the focus on breast cancer is not likely to reflect burden, but rather the high profile of this disease and the availability of specific funding for breast cancer research. This suggests a need for more effort toward examining and addressing evidence-practice gaps in care for other cancers with poor outcomes.

### Limitations

These results should be considered in light of several limitations. First, grey literature such as reports, policy documents, and dissertations were not included, nor were protocol papers. While this information may be relevant, grey literature is not peer-reviewed and therefore may not meet the high standards of quality associated with peer-reviewed publication. Inclusion of grey literature may also have biased the review given that papers related to work known by the authors and their network would have been more likely to have been identified than other works. Second, only one author screened the retrieved citations/abstracts. While a random 10% of included studies were double coded by a second reviewer with perfect agreement, this is a limitation of the search execution. Third, there are limitations to using volume of research output as a measure of research effort [36,38]. Due to publication bias, studies with unfavorable results may not be published, leading to under-representation of the true amount of work carried out in the field [39,40]. Finally, only studies that self-identified as addressing the evidence-practice gap in cancer care were included. While this may have resulted in some relevant studies not being included, it was not feasible to retrospectively define whether studies were addressing evidence-practice gaps. The use of a large number of search terms that covered evidence-based practice, guideline development, and the evidence-practice gap is likely to have limited the number of relevant studies missed. However, the large number of ways papers relating to evidence-practice gaps or implementation science are indexed is a limitation, compromising the ability of the field to advance in an optimal fashion.

## Conclusions

Reducing discrepancies between best evidence and clinical practice is an area of ongoing need across all fields of healthcare. A prerequisite for the effective transfer of evidence into practice is methodologically rigorous research that identifies where evidence-practice gaps exist, and develops and tests interventions to close the gap. The small number of intervention studies addressing evidence-practice gaps in cancer care highlights a clear need to shift research efforts from descriptive studies to robust experimental studies if patients are to receive optimal care. The relatively high number of studies on the translation of evidence into practice in breast cancer care suggests that greater research effort should be directed towards other cancers with high incidence and disease burden such as lung cancer.

## Competing interests

The authors declare that they have no competing interests.

## Authors’ contributions

JB and RSF conceived of and designed the review. JB, AB, KJ, and MC undertook data extraction. All authors contributed to drafting of the manuscript and have read and approved the final manuscript.

## Supplementary Material

Additional file 1: Table S1 Search Strategy. Click here for file

Additional file 2 Review Protocol. Click here for file

Additional file 3 List of citations for included studies. Click here for file
