# Supplementary material for: Examining and addressing evidence-practice gaps in cancer care: a systematic review
Source: Implement Sci. 2014 Mar 25;9:37. doi: 10.1186/1748-5908-9-37 (PMC4114221; doi:10.1186/1748-5908-9-37)
Supplement: Additional file 1: Table S1 — Search Strategy. [file 1748-5908-9-37-S1.docx]

**Additional file 1: Table S1 Search Strategy**

1. Guideline Adherence/

2. Evidence-Based Practice/

3. adherence.mp.

4. Compliance/

5. Adoption/

6. adoption.mp.

7. implementation.mp.

8. Practice guideline/

9. guideline/

10. guidelines.mp.

11. Evidence-Based Medicine/

12. translation.mp.

13. dissemination.mp.

14. exp Neoplasms/

15. oncology.mp.

16. cancer.mp.

17. Medical Oncology/

18. Radiation Oncology/

19. chemotherapy.mp.

20. Radiotherapy/

21. treatment.mp.

22. Diagnosis/

23. surveillance.mp.

24. 1 or 2 or 3 or 4 or 5 or 6 or 7 or 8 or 9 or 10 or 11 or 12 or 13

25. 14 or 15 or 16

26. 17 or 18 or 19 or 20 or 21 or 22 or 23

27. 24 and 25 and 26

28. limit 27 to (english language and humans)

29. limit 28 to yr="2000"

30. limit 28 to yr="2005"

31. limit 28 to yr="2010"
