# Supplementary material for: Examining and addressing evidence-practice gaps in cancer care: a systematic review
Source: Implement Sci. 2014 Mar 25;9:37. doi: 10.1186/1748-5908-9-37 (PMC4114221; doi:10.1186/1748-5908-9-37)
Supplement: Additional file 2 — Review Protocol. [file 1748-5908-9-37-S2.docx]

**Protocol:**

**Examining and addressing evidence-practice gaps in cancer care: a systematic review**

**Aim**

To examine in the cancer literature in the years 2000, 2005 and 2010:

1. The number of publications examining evidence-practice gaps;
2. The number of data-based versus non-data-based publications examining evidence-practice gaps;
3. The number of data-based publications examining evidence-practice gaps by (a) cancer type and (b) research design.

**Search Strategy**

The search strategy will be systematic. A list of Medical Subject Headings (Mesh) and keywords will be devised based on: (i) the author’s background reading; (ii) key words used to index known papers meeting eligibility criteria; and (ii) consultation with a medical librarian. Once finalized, the search will be conducted in Medline using the OVID platform. To ensure all relevant literature is identified, a Google Scholar search will be conducted using combinations of the keywords used in the Medline search. The first 100 results of each of these searches will be reviewed. The results of all searches will be imported into the reference management program EndNote for coding.

**Inclusion and exclusion criteria**

A priori inclusion and exclusion criteria will be developed and used to determine whether publications identified by the searches are included in the review. Inclusion and exclusion criteria will be refined as required during the literature searching process to ensure comprehensiveness. Articles will be assessed against inclusion and exclusion criteria in a two-phase process. Firstly, the primary author will review all search results and determine, based on the title and abstract, whether the study meets inclusion and exclusion criteria. These criteria will be applied twice: first at the title and abstract review stage, and a second time at the full-text article review stage. Studies that do not meet inclusion criteria after assessment of the full text article will be excluded with the reason for exclusion recorded. The following inclusion and exclusion criteria will be applied:

***Inclusion criteria***

- Published in English in 2000, 2005 and 2010;
- Report on evidence-practice gaps in relation to care for any type of cancer.

***Exclusion criteria***

- Papers examining the effectiveness of treatments on cancer recurrence, disease-free survival or overall survival;
- Studies examining evidence-practice gaps for cancer screening;
- Editorials, letters to the editor, dissertations and protocol papers.

**Diagram of search strategy and coding**

Development of search terms

Google Scholar search

Medline search

Initial assessment (title and abstract) against eligibility criteria by primary author

Secondary assessment using full text articles to determine eligibility

Coding according to: data based or non- data based; publication type (descriptive or intervention); cancer type

Double coding of random 10% of papers by co-author

**Coding**

Once eligible papers are identified, a coding process will be undertaken using the full-text manuscript. Coding will be carried out by the primary author, with a random 10% sample of included papers double coded by a second author to ensure accuracy. Any discrepancies will be resolved through discussion, or referral to another co-author for adjudication if a decision cannot be made.

Each paper will be coded as:

***Data-based or non-data-based.*** All publications identified as eligible will be coded as data based or non-data-based. Data-based publications will be defined as any publications that present primary data. Non data-based publications will be those which do not present primary data including: literature reviews; debates; commentary and opinion pieces.

***Publication type.*** Papers meeting eligibility criteria and coded as data-based will be further coded as:

- *Descriptive:* publications describing evidence practice gaps where data has been collected via self-report, survey, interview, focus group, observation. Includes interventions without baseline.
- *Intervention:* publications documenting an intervention that aims to reduce the evidence practice gap where the study design was a randomised controlled trial; controlled clinical trial; controlled before and after study; or interrupted time series.

**Cancer type.** Papers meeting eligibility criteria and coded as data-based will be further coded according to the cancer type of the study sample. The framework of the [International Classification of Diseases for Oncology](http://www.who.int/classifications/icd/adaptations/oncology/)[27] and body system-specific cancer classification will be used to classify cancer type.

**Outcomes**

The following outcomes will be reported:

- The number of search results returned by the searches;
- The number of papers meeting eligibility criteria by year;
- The number of papers meeting eligibility criteria by data based and non-data based by year;
- The number of papers meeting eligibility criteria by publication type by year;
- The number of papers meeting eligibility criteria by cancer type.
