# Supplementary material for: Examining and addressing evidence-practice gaps in cancer care: a systematic review
Source: Implement Sci. 2014 Mar 25;9:37. doi: 10.1186/1748-5908-9-37 (PMC4114221; doi:10.1186/1748-5908-9-37)
Supplement: Additional file 3 — List of citations for included studies. [file 1748-5908-9-37-S3.docx]

**Year: 2000**

**Descriptive**

1. Baker J, McCune JS, Harvey RD, 3rd, Bonsignore C, Lindley CM. Granulocyte colony-stimulating factor use in cancer patients. *Ann Pharmacother* 2000; **34**(7-8): 851-7.

2. Barton MB, Rose A, Lonergan D, Thornton D, O'Brien P, Trotter G. Mantle planning: report of the Australasian Radiation Oncology Lymphoma Group film survey and consensus guidelines. *Australas Radiol* 2000; **44**(4): 433-8.

3. Bickell NA, Aufses AH, Jr., Chassin MR. The quality of early-stage breast cancer care. *Ann Surg* 2000; **232**(2): 220-4.

4. Cooper GS, Yuan Z, Chak A, Rimm AA. Patterns of endoscopic follow-up after surgery for nonmetastatic colorectal cancer. *Gastrointest Endosc* 2000; **52**(1): 33-8.

5. Craft PS, Zhang Y, Brogan J, Tait N, Buckingham JM. Implementing clinical practice guidelines: a community-based audit of breast cancer treatment. Australian Capital Territory and South Eastern New South Wales Breast Cancer Treatment Group. *Med J Aust* 2000; **172**(5): 213-6.

6. Gard GB, Quinn MA, Narayan K, Bernshaw DM, Planner RS, Taylor M. Referral patterns for gynaecological radiotherapy in Victoria. *Aust N Z J Obstet Gynaecol* 2000; **40**(1): 62-5.

7. Graham ID, Evans WK, Logan D, et al. Canadian oncologists and clinical practice guidelines: a national survey of attitudes and reported use. Provincial Lung Disease Site Group of Cancer Care Ontario. *Oncology* 2000; **59**(4): 283-90.

8. Hokanson P, Seshadri R, Miller KD. Underutilization of breast-conserving therapy in a predominantly rural population: need for improved surgeon and public education. *Clin Breast Cancer* 2000; **1**(1): 72-6.

9. Holzer S, Reiners C, Mann K, et al. Patterns of care for patients with primary differentiated carcinoma of the thyroid gland treated in Germany during 1996. U.S. and German Thyroid Cancer Group. *Cancer* 2000; **89**(1): 192-201.

10. Howell E, Chen YT, Moradi M, Concato J. Cervical cancer practice patterns and appropriateness of therapy. *Am J Obstet Gynecol* 2000; **183**(2): 407-13.

11. Kitchen PR, Cawson JN, Krishnan CM, Barbetti TM, Henderson MA. Axillary dissection and ductal carcinoma in situ of the breast: a change in practice. *Aust N Z J Surg* 2000; **70**(6): 419-22.

12. Lash TL, Silliman RA, Guadagnoli E, Mor V. The effect of less than definitive care on breast carcinoma recurrence and mortality. *Cancer* 2000; **89**(8): 1739-47.

13. Levie NS, de Kraker J, Bokkerink JP, Appel IM, Aronson DC. SIOP treatment guidelines for renal tumours in small infants: fact or fantasy? *Eur J Surg Oncol* 2000; **26**(6): 567-70.

14. Mille D, Roy T, Carrere MO, et al. Economic impact of harmonizing medical practices: compliance with clinical practice guidelines in the follow-up of breast cancer in a French Comprehensive Cancer Center. *J Clin Oncol* 2000; **18**(8): 1718-24.

15. Nattinger AB, Hoffmann RG, Kneusel RT, Schapira MM. Relation between appropriateness of primary therapy for early-stage breast carcinoma and increased use of breast-conserving surgery. *Lancet* 2000; **356**(9236): 1148-53.

16. Pasztelyi Z, Schuler D, Czvenits E. Practice guidelines in pediatric hematooncology: implementation and survey. A possible way for medical quality assurance. *Pediatr Hematol Oncol* 2000; **17**(8): 679-85.

17. Richardson GE, Thursfield VJ, Giles GG. Reported management of lung cancer in Victoria in 1993: comparison with best practice. Anti-Cancer Council of Victoria Lung Cancer Study Group. *Med J Aust* 2000; **172**(7): 321-4.

18. Selwood K. Integrated care pathways: an audit tool in paediatric oncology. *Br J Nurs* 2000; **9**(1): 34-8.

19. Shank B, Moughan J, Owen J, Wilson F, Hanks GE. The 1993-94 patterns of care process survey for breast irradiation after breast-conserving surgery-comparison with the 1992 standard for breast conservation treatment. The Patterns of Care Study, American College of Radiology. *Int J Radiat Oncol Biol Phys* 2000; **48**(5): 1291-9.

20. Srisomboon J, Pantusart A, Phongnarisorn C, Suprasert P. Reasons for improper simple hysterectomy in patients with invasive cervical cancer in the northern region of Thailand. *J Obstet Gynaecol Res* 2000; **26**(3): 175-80.

21. Swanson G, Bergstrom K, Stump E, Miyahara T, Herfindal ET. Growth factor usage patterns and outcomes in the community setting: collection through a practice-based computerized clinical information system. *J Clin Oncol* 2000; **18**(8): 1764-70.

**Intervention**

22. Bell CM, Ma M, Campbell S, Basnett I, Pollock A, Taylor I. Methodological issues in the use of guidelines and audit to improve clinical effectiveness in breast cancer in one United Kingdom health region. *Eur J Surg Oncol* 2000; **26**(2): 130-6.

23. Du Pen AR, Du Pen S, Hansberry J, et al. An educational implementation of a cancer pain algorithm for ambulatory care. *Pain Manag Nurs* 2000; **1**(4): 116-28.

24. Mor V, Laliberte LL, Petrisek AC, et al. Impact of breast cancer treatment guidelines on surgeon practice patterns: results of a hospital-based intervention. *Surgery* 2000; **128**(5): 847-61.

**Review**

25. Bookman MA. Using tumor registry resources in analyzing concordance with guidelines and outcomes. *Oncology (Williston)* 2000; **14**(11A): 104-7.

**Year: 2005**

**Descriptive**

26. Aarts MC, Hogen Esch TT, Terhaard CH, Koole R, Hordijk GJ. Guidelines in head and neck oncology compliance and consequences of deviations from the standard protocol for tongue and floor of mouth squamous carcinoma. *Clin Otolaryngol* 2005; **30**(5): 444-50.

27. Akhtar SS, Nadrah HM. Assessment of the quality of breast cancer care: a single institutional study from Saudi Arabia. *Int J Qual Health Care* 2005; **17**(4): 301-5.

28. Biermann M, Pixberg MK, Dorr U, et al. Guidelines on radioiodine therapy for differentiated thyroid carcinoma: impact on clinical practice. *Nucl Med (Stuttg)* 2005; **44**(6): 229-34, 36-7.

29. Boulin M, Lejeune C, Le Teuff G, et al. Patterns of surveillance practices after curative surgery for colorectal cancer in a French population. *Dis Colon Rectum* 2005; **48**(10): 1890-9.

30. Brouwer J, Bree R, Hoekstra OS, Langendijk JA, Castelijns JA, Leemans CR. Screening for distant metastases in patients with head and neck cancer: what is the current clinical practice? *Clin Otolaryngol* 2005; **30**(5): 438-43.

31. Buzdar A, Macahilig C. How rapidly do oncologists respond to clinical trial data? *Oncologist* 2005; **10**(1): 15-21.

32. Chamberlain JD, Smibert E, Skeen J, Alvaro F. Prospective audit of treatment of paediatric febrile neutropenia in Australasia. *J Paediatr Child Health* 2005; **41**(11): 598-603.

33. Cormier JN, Xing Y, Ding M, et al. Population-based assessment of surgical treatment trends for patients with melanoma in the era of sentinel lymph node biopsy. *J Clin Oncol* 2005; **23**(25): 6054-62.

34. Danova M, Rosti G, De Placido S, Bencardino K, Venturini M. Use of granulocyte colony-stimulating factor: a survey among Italian medical oncologists. *Oncol Rep* 2005; **14**(6): 1405-12.

35. Delaney G, Jacob S, Barton M. Estimation of an optimal external beam radiotherapy utilization rate for head and neck carcinoma. *Cancer* 2005; **103**(11): 2216-27.

36. Deo SV, Samaiya A, Shukla NK, et al. Breast conservation therapy for breast cancer: patient profile and treatment outcome at a tertiary care cancer centre. *Natl Med J India* 2005; **18**(4): 178-81.

37. Dranitsaris G, Evans WK, Milliken D, Zanke B. The impact of practice guidelines and funding policies on the use of new drugs in advanced non-small cell lung cancer. *J Eval Clin Pract* 2005; **11**(4): 350-6.

38. Drug Utilization Review Team in O. Adjuvant systemic therapies in patients with colorectal cancer: an audit on clinical practice in Italy. *Tumori* 2005; **91**(6): 472-6.

39. Drummond R, Power A, Evans A, et al. Changes in practice of breast cancer radiotherapy 1998-2002: an Australasian survey. *Australas Radiol* 2005; **49**(1): 44-52.

40. Elston Lafata J, Simpkins J, Schultz L, et al. Routine surveillance care after cancer treatment with curative intent. *Med Care* 2005; **43**(6): 592-9.

41. Engel J, Kerr J, Eckel R, et al. Quality of treatment in routine care in a population sample of rectal cancer patients. *Acta Oncol* 2005; **44**(1): 65-74.

42. Haas JS, Kaplan CP, Brawarsky P, Kerlikowske K. Evaluation and outcomes of women with a breast lump and a normal mammogram result. *J Gen Intern Med* 2005; **20**(8): 692-6.

43. Hachiya T, Akakura K, Saito S, et al. A retrospective study of the treatment of locally advanced prostate cancer by six institutions in eastern and north-eastern Japan. *BJU Int* 2005; **95**(4): 534-40.

44. Harlan LC, Greene AL, Clegg LX, Mooney M, Stevens JL, Brown ML. Insurance status and the use of guideline therapy in the treatment of selected cancers. *J Clin Oncol* 2005; **23**(36): 9079-88.

45. Issell BF, Maskarinec G, Pagano I, Gotay CC. Breast cancer treatment among women of different ethnicity in Hawaii. *Cancer Invest* 2005; **23**(6): 497-504.

46. Jestin P, Pahlman L, Glimelius B, Gunnarsson U. Cancer staging and survival in colon cancer is dependent on the quality of the pathologists' specimen examination. *Eur J Cancer* 2005; **41**(14): 2071-8.

47. Katz SJ, Lantz PM, Janz NK, et al. Surgeon perspectives about local therapy for breast carcinoma. *Cancer* 2005; **104**(9): 1854-61.

48. Kebebew E, Greenspan FS, Clark OH, Woeber KA, Grunwell J. Extent of disease and practice patterns for medullary thyroid cancer. *J Am Coll Surg* 2005; **200**(6): 890-6.

49. Korner H, Soreide K, Stokkeland PJ, Soreide JA. Systematic follow-up after curative surgery for colorectal cancer in Norway: a population-based audit of effectiveness, costs, and compliance. *J Gastrointest Surg* 2005; **9**(3): 320-8.

50. Koshy A, Buckingham JM, Zhang Y, et al. Surgical management of invasive breast cancer: a 5-year prospective study of treatment in the Australian Capital Territory and South-Eastern New South Wales. *ANZ J Surg* 2005; **75**(9): 757-61.

51. Laliberte L, Fennell ML, Papandonatos G. The relationship of membership in research networks to compliance with treatment guidelines for early-stage breast cancer. *Med Care* 2005; **43**(5): 471-9.

52. Langer CJ, Moughan J, Movsas B, et al. Patterns of care survey (PCS) in lung cancer: how well does current U.S. practice with chemotherapy in the non-metastatic setting follow the literature? *Lung Cancer* 2005; **48**(1): 93-102.

53. Luke CG, Koczwara B, Moore JE, et al. Treatment and survival from colorectal cancer: the experience of patients at South Australian teaching hospitals between 1980 and 2002. *Clin Oncol (R Coll Radiol)* 2005; **17**(5): 372-81.

54. Maas HA, Kruitwagen RF, Lemmens VE, Goey SH, Janssen-Heijnen ML. The influence of age and co-morbidity on treatment and prognosis of ovarian cancer: a population-based study. *Gynecol Oncol* 2005; **97**(1): 104-9.

55. Neuss MN, Desch CE, McNiff KK, et al. A process for measuring the quality of cancer care: the Quality Oncology Practice Initiative. *J Clin Oncol* 2005; **23**(25): 6233-9.

56. Nielsen HM, Overgaard J, Grau C, Christensen JJ, Overgaard M. Audit of the radiotherapy in the DBCG 82 b&c trials--a validation study of the 1,538 patients randomised to postmastectomy radiotherapy. *Radiother Oncol* 2005; **76**(3): 285-92.

57. Nystedt KE, Hill JE, Mitchell AM, et al. The standardization of radiation skin care in British Columbia: a collaborative approach. *Oncol Nurs Forum* 2005; **32**(6): 1199-205.

58. Ong S, Watters JM, Grunfeld E, O'Rourke K. Predictors of referral for adjuvant therapy for colorectal cancer. *Can J Surg* 2005; **48**(3): 225-9.

59. Razdan S, Johannes J, Cox M, Bagley DH. Current practice patterns in urologic management of upper-tract transitional-cell carcinoma. *J Endourol* 2005; **19**(3): 366-71.

60. Schaapveld M, de Vries EG, Otter R, de Vries J, Dolsma WV, Willemse PH. Guideline adherence for early breast cancer before and after introduction of the sentinel node biopsy. *Br J Cancer* 2005; **93**(5): 520-8.

61. Stahl M, Schweers K, Muller C, Koster W, Wilke H. Application of adjuvant chemotherapy in colorectal cancer -- a survey in the region of Essen, Germany. *Onkologie* 2005; **28**(1): 7-10.

62. Vulto JC, Louwman WJ, Poortmans PM, Coebergh JW. Hospital variation in referral for primary radiotherapy in South Netherlands, 1988-1999. *Eur J Cancer* 2005; **41**(17): 2722-7.

63. Wood K. Audit of nutritional guidelines for head and neck cancer patients undergoing radiotherapy. *J Hum Nutr Diet* 2005; **18**(5): 343-51.

64. de Roos MA, de Bock GH, Baas PC, de Munck L, Wiggers T, de Vries J. Compliance with guidelines is related to better local recurrence-free survival in ductal carcinoma in situ. *Br J Cancer* 2005; **93**(10): 1122-7.

65. Featherstone C, Delaney G, Jacob S, Barton M. Estimating the optimal utilization rates of radiotherapy for hematologic malignancies from a review of the evidence: part II-leukemia and myeloma. *Cancer* 2005; **103**(2): 393-401.

66. van Agthoven M, Heule-Dieleman HA, de Boer MF, et al. Evaluating adherence to the Dutch guideline for diagnosis, treatment and follow-up of laryngeal carcinomas. *Radiother Oncol* 2005; **74**(3): 337-44.

**Intervention**

1. Cleeland CS, Portenoy RK, Rue M, et al. Does an oral analgesic protocol improve pain control for patients with cancer? An intergroup study coordinated by the Eastern Cooperative Oncology Group. *Ann Oncol* 2005; **16**(6): 972-80.
2. Kedikoglou S, Syrigos K, Skalkidis Y, Ploiarchopoulou F, Dessypris N, Petridou E. Implementing clinical protocols in oncology: quality gaps and the learning curve phenomenon. *Eur J Public Health* 2005; **15**(4): 368-71.
3. Ray-Coquard I, Philip T, de Laroche G, et al. Persistence of medical change at implementation of clinical guidelines on medical practice: a controlled study in a cancer network. *J Clin Oncol* 2005; **23**(19): 4414-23.

**Review**

1. Albain KS, de la Garza Salazar J, Pienkowski T, et al. Reducing the global breast cancer burden: the importance of patterns of care research. *Clin Breast Cancer* 2005; **6**(5): 412-20.
2. Bickell NA, Mendez J, Guth AA. The quality of early-stage breast cancer treatment: what can we do to improve? *Surg Oncol Clin N Am* 2005; **14**(1): 103-17, vi.
3. Hurd TC, James T, Foster JM. Factors that affect breast cancer treatment: underserved and minority populations. *Surg Oncol Clin N Am* 2005; **14**(1): 119-30, vii.
4. Kaiser R. Antiemetic guidelines: are they being used? *Lancet Oncol* 2005; **6**(8): 622-5.
5. Ottevanger PB, De Mulder PH. The quality of chemotherapy and its quality assurance. *Eur J Surg Oncol* 2005; **31**(6): 656-66.
6. Smyth D, Zumbrink S. Optimising the management of anaemia in patients with cancer with practice guidelines using erythropoiesis-stimulating proteins. *Eur J Oncol Nurs* 2005; **9 Suppl 1**: S3-S13.
7. White N, Maxwell C, Michelson J, Bedell C. Protocols for managing chemotherapy-induced neutropenia in clinical oncology practices. *Cancer Nurs* 2005; **28**(1): 62-9.

**Year: 2010**

**Descriptive**

1. Akbar RA, Gosh SK, Khalil S, ul Haq SM. Zoledronic acid in metastatic bone disease: an audit based discussion. *J Ayub Med Coll Abbottabad* 2010; **22**(3): 5-7.
2. Allemani C, Storm H, Voogd AC, et al. Variation in 'standard care' for breast cancer across Europe: a EUROCARE-3 high resolution study. *Eur J Cancer* 2010; **46**(9): 1528-36.
3. Andre S, Taboulet P, Elie C, et al. Febrile neutropenia in French emergency departments: results of a prospective multicentre survey. *Crit Care* 2010; **14**(2): R68.
4. Auerbach AD, Maselli J, Carter J, Pekow PS, Lindenauer PK. The relationship between case volume, care quality, and outcomes of complex cancer surgery. *J Am Coll Surg* 2010; **211**(5): 601-8.
5. Augestad KM, Lindsetmo RO, Stulberg J, et al. International preoperative rectal cancer management: staging, neoadjuvant treatment, and impact of multidisciplinary teams. *World J Surg* 2010; **34**(11): 2689-700.
6. Bate J, Patel SR, Chisholm J, Heath PT, Supportive Care Group of the Children's C, Leukaemia G. Immunisation practices of paediatric oncology and shared care oncology consultants: a United Kingdom survey. *Pediatr Blood Cancer* 2010; **54**(7): 941-6.
7. Bayles AC, Sethia KK. The impact of Improving Outcomes Guidance on the management and outcomes of patients with carcinoma of the penis. *Ann R Coll Surg Engl* 2010; **92**(1): 44-5.
8. Berry J, Caplan L, Davis S, et al. A black-white comparison of the quality of stage-specific colon cancer treatment. *Cancer* 2010; **116**(3): 713-22.
9. Blessing JA, Bialy SA, Whitney CW, Stonebraker BL, Stehman FB. Gynecologic Oncology Group quality assurance audits: analysis and initiatives for improvement. *Clin* 2010; **7**(4): 390-9.
10. Booth CM, Shepherd FA, Peng Y, et al. Adoption of adjuvant chemotherapy for non-small-cell lung cancer: a population-based outcomes study. *J Clin Oncol* 2010; **28**(21): 3472-8.
11. Boulet S, Tixier H, Fraisse J, et al. Sentinel lymph node biopsy in two Burgundy districts: prospective multicentric study on 528 breast cancers during the year 2005. *Arch Gynecol Obstet* 2010; **281**(3): 491-8.
12. Caudron A, Chaby G, Dadban A, et al. Multidisciplinary team meetings in Oncology: first analysis of benefits and evaluation of activity in a Dermatology unit in France. *Eur J Dermatol* 2010; **20**(6): 778-84.
13. Cazap E, Buzaid A, Garbino C, et al. Breast cancer in Latin America: experts perceptions compared with medical care standards. *Breast* 2010; **19**(1): 50-4.
14. Cheng CW, Fan W, Ko SG, Song L, Bian ZX. Evidence-based management of herb-drug interaction in cancer chemotherapy. *Explore (NY)* 2010; **6**(5): 324-9.
15. Chivers K, Basnyat P, Taffinder N. The impact of national guidelines on the waiting list for colonoscopy: a quantitative clinical audit. *Colorectal Dis* 2010; **12**(7): 632-9.
16. Clavel S, Roy I, Carrier JF, Rousseau P, Fortin MA. Adjuvant regional irradiation after breast-conserving therapy for early stage breast cancer: a survey of canadian radiation oncologists. *Clin Oncol (R Coll Radiol)* 2010; **22**(1): 39-45.
17. Cooperberg MR, Broering JM, Carroll PR. Time trends and local variation in primary treatment of localized prostate cancer. *J Clin Oncol* 2010; **28**(7): 1117-23.
18. Coulson SG, Kumar VS, Manifold IM, et al. Review of testing and use of adjuvant trastuzumab across a cancer network--are we treating the right patients? *Clin Oncol (R Coll Radiol)* 2010; **22**(4): 289-93.
19. Craft PS, Buckingham JM, Dahlstrom JE, et al. Variation in the management of early breast cancer in rural and metropolitan centres: implications for the organisation of rural cancer services. *Breast* 2010; **19**(5): 396-401.
20. Crawford NW, Heath JA, Ashley D, Downie P, Buttery JP. Survivors of childhood cancer: an Australian audit of vaccination status after treatment. *Pediatr Blood Cancer* 2010; **54**(1): 128-33.
21. DeMartini WB, Ichikawa L, Yankaskas BC, et al. Breast MRI in community practice: equipment and imaging techniques at facilities in the Breast Cancer Surveillance Consortium. *Journal of the American College of Radiology* 2010; **7**(11): 878-84.
22. Devbhandari MP, Joshi V, Barber P, Krysiak P, Shah R, Jones MT. Active treatment rates for lung cancer in south Manchester: are we doing enough? *Interact Cardiovasc Thorac Surg* 2010; **11**(4): 411-4.
23. Donato BM, Burns L, Willey V, Cohenuram M, Oliveria S, Yood MU. Treatment patterns in patients with advanced breast cancer who were exposed to an anthracycline, a taxane, and capecitabine: a descriptive report. *Clin Ther* 2010; **32**(3): 546-54.
24. Elferink MA, Krijnen P, Wouters MW, et al. Variation in treatment and outcome of patients with rectal cancer by region, hospital type and volume in the Netherlands. *Eur J Surg Oncol* 2010; **36 Suppl 1**: S74-82.
25. Falandry C, Campone M, Cartron G, Guerin D, Freyer G. Trends in G-CSF use in 990 patients after EORTC and ASCO guidelines. *Eur J Cancer* 2010; **46**(13): 2389-98.
26. Famakinwa OM, Roman SA, Wang TS, Sosa JA. ATA practice guidelines for the treatment of differentiated thyroid cancer: were they followed in the United States? *Am J Surg* 2010; **199**(2): 189-98.
27. Farrington M, Cullen L, Dawson C. Assessment of oral mucositis in adult and pediatric oncology patients: an evidence-based approach. *ORL Head Neck Nurs* 2010; **28**(3): 8-15.
28. Fong A, Ng W, Barton MB, Delaney GP. Estimation of an evidence-based benchmark for the optimal endocrine therapy utilization rate in breast cancer. *Breast* 2010; **19**(5): 345-9.
29. Gaertner J, Wolf J, Ostgathe C, et al. Specifying WHO recommendation: moving toward disease-specific guidelines. *J Palliat Med* 2010; **13**(10): 1273-6.
30. Gatta G, Zigon G, Aareleid T, et al. Patterns of care for European colorectal cancer patients diagnosed 1996-1998: a EUROCARE high resolution study. *Acta Oncol* 2010; **49**(6): 776-83.
31. Gore JL, Litwin MS, Lai J, et al. Use of radical cystectomy for patients with invasive bladder cancer. *J Natl Cancer Inst* 2010; **102**(11): 802-11.
32. Gridelli C, Rossi A, de Marinis F. Pattern of care for advanced non-small cell lung cancer in the era of histology-based treatment: a survey of the Italian Association of Thoracic Oncology (AIOT). *Lung Cancer* 2010; **67**(3): 339-42.
33. Hancke K, Denkinger MD, Konig J, et al. Standard treatment of female patients with breast cancer decreases substantially for women aged 70 years and older: a German clinical cohort study. *Ann Oncol* 2010; **21**(4): 748-53.
34. Harlan LC, Zujewski JA, Goodman MT, Stevens JL. Breast cancer in men in the United States: a population-based study of diagnosis, treatment, and survival. *Cancer* 2010; **116**(15): 3558-68.
35. Hayman AV, Chang ET, Molokie RE, Kahng LS, Prystowsky JB, Bentrem DJ. Assessing compliance with national quality measures to improve colorectal cancer care at the VA. *Am J Surg* 2010; **200**(5): 572-6.
36. Henderson TO, Hlubocky FJ, Wroblewski KE, Diller L, Daugherty CK. Physician preferences and knowledge gaps regarding the care of childhood cancer survivors: a mailed survey of pediatric oncologists. *J Clin Oncol* 2010; **28**(5): 878-83.
37. Hernes E, Kyrdalen A, Kvale R, et al. Initial management of prostate cancer: first year experience with the Norwegian National Prostate Cancer Registry. *BJU Int* 2010; **105**(6): 805-11; discussion 11.
38. Herr K, Titler M, Fine P, et al. Assessing and treating pain in hospices: current state of evidence-based practices. *J Pain Symptom Manage* 2010; **39**(5): 803-19.
39. Hessel AC, Moreno MA, Hanna EY, et al. Compliance with quality assurance measures in patients treated for early oral tongue cancer. *Cancer* 2010; **116**(14): 3408-16.
40. Hollowell K, Olmsted CL, Richardson AS, et al. American Society of Clinical Oncology-recommended surveillance and physician specialty among long-term breast cancer survivors. *Cancer* 2010; **116**(9): 2090-8.
41. Holmebakk T, Frykholm G, Viste A, Norwegian Gastrointestinal Cancer G. Introducing national guidelines on perioperative chemotherapy for gastric cancer in Norway: a retrospective audit. *Eur J Surg Oncol* 2010; **36**(7): 610-6.
42. Jackson GL, Melton LD, Abbott DH, et al. Quality of nonmetastatic colorectal cancer care in the Department of Veterans Affairs.[Erratum appears in J Clin Oncol. 2010 Aug 10;28(23):3797]. *J Clin Oncol* 2010; **28**(19): 3176-81.
43. Jacob S, Hovey E, Ng W, Vinod S, Delaney GP, Barton MB. Estimation of an optimal chemotherapy utilisation rate for lung cancer: an evidence-based benchmark for cancer care. *Lung Cancer* 2010; **69**(3): 307-14.
44. Jacob S, Wong K, Delaney GP, Adams P, Barton MB. Estimation of an optimal utilisation rate for palliative radiotherapy in newly diagnosed cancer patients. *Clin Oncol (R Coll Radiol)* 2010; **22**(1): 56-64.
45. Jeevan R, Cromwell DA, Browne JP, et al. Regional variation in use of immediate breast reconstruction after mastectomy for breast cancer in England. *Eur J Surg Oncol* 2010; **36**(8): 750-5.
46. Jones C, Badger SA, McClements J, McKie L, Diamond T, Taylor MA. Can the National Health Service Cancer Plan timeline be applied to colorectal hepatic metastases? *Ann R Coll Surg Engl* 2010; **92**(2): 136-8.
47. Kapp JM, Walker R, Haneuse S, Buist DS, Yankaskas BC. Are there racial/ethnic disparities among women younger than 40 undergoing mammography? *Breast Cancer Res Treat* 2010; **124**(1): 213-22.
48. Kurtz JE, Heitz D, Serra S, et al. Adjuvant chemotherapy in elderly patients with colorectal cancer. A retrospective analysis of the implementation of tumor board recommendations in a single institution. *Crit Rev Oncol Hematol* 2010; **74**(3): 211-7.
49. Lewis CM, Hessel AC, Roberts DB, et al. Prereferral head and neck cancer treatment: compliance with national comprehensive cancer network treatment guidelines. *Arch Otolaryngol Head Neck Surg* 2010; **136**(12): 1205-11.
50. Malin JL, Diamant AL, Leake B, et al. Quality of care for breast cancer for uninsured women in california under the breast and cervical cancer prevention treatment act. *J Clin Oncol* 2010; **28**(21): 3479-84.
51. Mayo SC, Shore AD, Nathan H, et al. National trends in the management and survival of surgically managed gallbladder adenocarcinoma over 15 years: a population-based analysis. *J Gastrointest Surg* 2010; **14**(10): 1578-91.
52. McBride RB, Lebwohl B, Hershman DL, Neugut AI. Impact of socioeconomic status on extent of lymph node dissection for colon cancer. *Cancer Epidemiol Biomarkers Prev* 2010; **19**(3): 738-45.
53. Nakhleh RE, Grimm EE, Idowu MO, Souers RJ, Fitzgibbons PL. Laboratory compliance with the American Society of Clinical Oncology/college of American Pathologists guidelines for human epidermal growth factor receptor 2 testing: a College of American Pathologists survey of 757 laboratories. *Arch Pathol Lab Med* 2010; **134**(5): 728-34.
54. Nirenberg A, Reame NK, Cato KD, Larson EL. Oncology nurses' use of National Comprehensive Cancer Network clinical practice guidelines for chemotherapy-induced and febrile neutropenia. *Oncol Nurs Forum* 2010; **37**(6): 765-73.
55. Owen-Smith A, Coast J, Donovan J. The usefulness of NICE guidance in practice: different perspectives of managers, clinicians, and patients. *Int J Technol Assess Health Care* 2010; **26**(3): 317-22.
56. Panigrahi B, Roman SA, Sosa JA. Medullary thyroid cancer: are practice patterns in the United States discordant from American Thyroid Association guidelines? *Ann Surg Oncol* 2010; **17**(6): 1490-8.
57. Park HS, Roman SA, Sosa JA. Treatment patterns of aging Americans with differentiated thyroid cancer. *Cancer* 2010; **116**(1): 20-30.
58. Phang PT, McGahan CE, McGregor G, et al. Effects of change in rectal cancer management on outcomes in British Columbia. *Can J Surg* 2010; **53**(4): 225-31.
59. Pisu M, Richardson LC, Kim YI, et al. Less-than-standard treatment in rectal cancer patients: which patients are at risk? *J Natl Med Assoc* 2010; **102**(3): 190-8.
60. Raine R, Wong W, Scholes S, Ashton C, Obichere A, Ambler G. Social variations in access to hospital care for patients with colorectal, breast, and lung cancer between 1999 and 2006: retrospective analysis of hospital episode statistics. *Bmj* 2010; **340**: b5479.
61. Ramsey SD, McCune JS, Blough DK, et al. Colony-stimulating factor prescribing patterns in patients receiving chemotherapy for cancer. *Am J Manag Care* 2010; **16**(9): 678-86.
62. Richardson LC, Royalty J, Howe W, Helsel W, Kammerer W, Benard VB. Timeliness of breast cancer diagnosis and initiation of treatment in the National Breast and Cervical Cancer Early Detection Program, 1996-2005. *Am J Public Health* 2010; **100**(9): 1769-76.
63. Rogers SO, Jr., Gray SW, Landrum MB, et al. Variations in surgeon treatment recommendations for lobectomy in early-stage non-small-cell lung cancer by patient age and comorbidity. *Ann Surg Oncol* 2010; **17**(6): 1581-8.
64. Salz T, Weinberger M, Ayanian JZ, et al. Variation in use of surveillance colonoscopy among colorectal cancer survivors in the United States. *BMC Health Serv Res* 2010; **10**: 256.
65. Selwood K, Ward E, Gibson F. Assessment and management of nutritional challenges in children's cancer care: a survey of current practice in the United Kingdom. *Eur J Oncol Nurs* 2010; **14**(5): 439-46.
66. Simons PA, Houben RM, Backes HH, Pijls RF, Groothuis S. Compliance to technical guidelines for radiotherapy treatment in relation to patient safety. *Int J Qual Health Care* 2010; **22**(3): 187-93.
67. Tanaka N, Katai H, Taniguchi H, et al. Trends in characteristics of surgically treated early gastric cancer patients after the introduction of gastric cancer treatment guidelines in Japan. *Gastric Cancer* 2010; **13**(2): 74-7.
68. van Dyk S, Byram D, Bernshaw D. Use of 3D imaging and awareness of GEC-ESTRO recommendations for cervix cancer brachytherapy throughout Australia and New Zealand. *J Med Imaging Radiat Oncol* 2010; **54**(4): 383-7.
69. van Nes JG, Seynaeve C, Jones S, et al. Variations in locoregional therapy in postmenopausal patients with early breast cancer treated in different countries. *Br J Surg* 2010; **97**(5): 671-9.
70. van Nes JG, Seynaeve C, Maartense E, et al. Patterns of care in Dutch postmenopausal patients with hormone-sensitive early breast cancer participating in the Tamoxifen Exemestane Adjuvant Multinational (TEAM) trial. *Ann Oncol* 2010; **21**(5): 974-82.
71. van Steenbergen LN, Rutten HJ, Creemers GJ, Pruijt JF, Coebergh JW, Lemmens VE. Large age and hospital-dependent variation in administration of adjuvant chemotherapy for stage III colon cancer in southern Netherlands. *Ann Oncol* 2010; **21**(6): 1273-8.
72. van Steenbergen LN, van de Poll-Franse LV, Wouters MW, et al. Variation in management of early breast cancer in the Netherlands, 2003-2006. *Eur J Surg Oncol* 2010; **36 Suppl 1**: S36-43.
73. Viehl CT, Ochsner A, von Holzen U, et al. Inadequate quality of surveillance after curative surgery for colon cancer. *Ann Surg Oncol* 2010; **17**(10): 2663-9.
74. Vinod SK, Sidhom MA, Gabriel GS, Lee MT, Delaney GP. Why do some lung cancer patients receive no anticancer treatment? *J Thorac Oncol* 2010; **5**(7): 1025-32.
75. Wasif N, Maggard MA, Ko CY, Giuliano AE. Underuse of axillary dissection for the management of sentinel node micrometastases in breast cancer. *Arch Surg* 2010; **145**(2): 161-6.
76. White V, Pruden M, Kitchen P, Villanueva E, Erbas B. The impact of publication of Australian treatment recommendations for DCIS on clinical practice: A population-based, "before-after" study. *Eur J Surg Oncol* 2010; **36**(10): 949-56.
77. Wilke LG, Ballman KV, McCall LM, et al. Adherence to the National Quality Forum (NQF) breast cancer measures within cancer clinical trials: a review from ACOSOG Z0010. *Ann Surg Oncol* 2010; **17**(8): 1989-94.
78. Winget M, Hossain S, Yasui Y, Scarfe A. Characteristics of patients with stage III colon adenocarcinoma who fail to receive guideline-recommended treatment. *Cancer* 2010; **116**(20): 4849-56.
79. Wockel A, Varga D, Atassi Z, et al. Impact of guideline conformity on breast cancer therapy: results of a 13-year retrospective cohort study. *Onkologie* 2010; **33**(1-2): 21-8.
80. Wouters MW, Siesling S, Jansen-Landheer ML, et al. Variation in treatment and outcome in patients with non-small cell lung cancer by region, hospital type and volume in the Netherlands. *Eur J Surg Oncol* 2010; **36 Suppl 1**: S83-92.
81. Yee SS, Dutta PR, Solin LJ, Vapiwala N, Kao GD. Lack of compliance with national vaccination guidelines in oncology patients receiving radiation therapy. *J Support Oncol* 2010; **8**(1): 28-34.
82. Yu X, McBean AM. Screening mammography use and chemotherapy among female stage II colon cancer patients: a retrospective cohort study. *BMC Health Serv Res* 2010; **10**: 98.
83. Escobar Alvarez Y, Rodriguez Sanchez CA, Caballero Martinez F, Recuero Cuervo V, Camps Herrero C. Professional survey on knowledge and clinical patterns of pain management in Spanish medical oncology. *Clin Transl Oncol* 2010; **12**(12): 819-24.
84. Lwu S, Hamilton MG, Forsyth PA, Cairncross JG, Parney IF. Use of peri-operative anti-epileptic drugs in patients with newly diagnosed high grade malignant glioma: a single center experience. *J Neurooncol* 2010; **96**(3): 403-8.
85. Pagano L, Caira M, Offidani M, et al. Adherence to international guidelines for the treatment of invasive aspergillosis in acute myeloid leukaemia: feasibility and utility (SEIFEM-2008B study). *J Antimicrob Chemother* 2010; **65**(9): 2013-8.
86. Pantaleo MA, Di Battista M, La Rovere S, et al. Management of patients with gastrointestinal stromal tumor in clinical practice in Italy: a critical "event tree model" analysis of decision-making processes and outcomes. *Tumori* 2010; **96**(2): 219-28.
87. Schlenker B, Seitz M, Bader MJ, et al. Comparison of guideline recommendations with daily practice in patients with renal cell carcinoma. *Eur J Med Res* 2010; **15**(6): 253-7.
88. Wu AW, Wang MB, Nguyen CT. Surgical practice patterns in the treatment of papillary thyroid microcarcinoma.[Erratum appears in Arch Otolaryngol Head Neck Surg. 2011 Feb;137(2):123]. *Arch Otolaryngol Head Neck Surg* 2010; **136**(12): 1182-90.

**Intervention**

1. Freeman RK, Van Woerkom JM, Vyverberg A, Ascioti AJ. The effect of a multidisciplinary thoracic malignancy conference on the treatment of patients with lung cancer. *Eur J Cardiothorac Surg* 2010; **38**(1): 1-5.
2. Frenzel JC, Kee SS, Ensor JE, Riedel BJ, Ruiz JR. Ongoing provision of individual clinician performance data improves practice behavior. *Anesth Analg* 2010; **111**(2): 515-9.
3. Schrader M, Weissbach L, Hartmann M, et al. Burden or relief: do second-opinion centers influence the quality of care delivered to patients with testicular germ cell cancer? *Eur Urol* 2010; **57**(5): 867-72.
4. Van Erps J, Aapro M, MacDonald K, et al. Promoting evidence-based management of anemia in cancer patients: concurrent and discriminant validity of RESPOND, a web-based clinical guidance system based on the EORTC guidelines for supportive care in cancer. *Support Care Cancer* 2010; **18**(7): 847-58.

**Review**

1. Akaza H. Future prospects for luteinizing hormone-releasing hormone analogues in prostate cancer treatment. *Pharmacology* 2010; **85**(2): 110-20.
2. Barocas DA, Penson DF. Racial variation in the pattern and quality of care for prostate cancer in the USA: mind the gap. *BJU Int* 2010; **106**(3): 322-8.
3. Bernier J. Translational breast cancer research: recent advances through the lens of experimental radiotherapy. *Breast* 2010; **19**(1): 23-7.
4. Fairchild A. Under-treatment of cancer pain. *Current Opinion in Supportive & Palliative Care* 2010; **4**(1): 11-5.
5. Farge D, Durant C, Villiers S, et al. Lessons from French National Guidelines on the treatment of venous thrombosis and central venous catheter thrombosis in cancer patients. *Thromb Res* 2010; **125 Suppl 2**: S108-16.
6. Gluck S, Mamounas T. Improving outcomes in early-stage breast cancer. *Oncology (Williston)* 2010; **24**(11 Suppl 4): 1-15.
7. Simon R. Translational research in oncology: key bottlenecks and new paradigms. *Expert Rev Mol Med* 2010; **12**: e32.
8. Van Ryckeghem F, Van Belle S. Management of chemotherapy-induced nausea and vomiting. *Acta Clin Belg* 2010; **65**(5): 305-10.
